# Markov Chain Wave Generative Adversarial Network for Bee Bioacoustic Signal Synthesis

**DOI:** 10.3390/s26020371

**Published:** 2026-01-06

**Authors:** Kumudu Samarappuli, Iman Ardekani, Mahsa Mohaghegh, Abdolhossein Sarrafzadeh

**Affiliations:** 1 School of Computing, Electrical Engineering and Applied Technology, Unitec Institute of Technology, Auckland 1025, New Zealand; samark02@myunitec.ac.nz; 2 School of Arts and Sciences, The University of Notre Dame Australia (Sydney Campus), Darlinghurst, NSW 2007, Australia; 3 School of Engineering, Computer and Mathematical Sciences, Auckland University of Technology, Auckland 1010, New Zealand; mahsa.mohaghegh@aut.ac.nz; 4 Batten College of Engineering and Technology, Old Dominion University, Norfolk, VA 23529, USA; asarrafz@odu.edu

**Keywords:** bee bioacoustic, synthetic data, generative adversarial networks, Markov Chain, smart beekeeping

## Abstract

This paper presents a framework for synthesizing bee bioacoustic signals associated with hive events. While existing approaches like WaveGAN have shown promise in audio generation, they often fail to preserve the subtle temporal and spectral features of bioacoustic signals critical for event-specific classification. The proposed method, MCWaveGAN, extends WaveGAN with a Markov Chain refinement stage, producing synthetic signals that more closely match the distribution of real bioacoustic data. Experimental results show that this method captures signal characteristics more effectively than WaveGAN alone. Furthermore, when integrated into a classifier, synthesized signals improved hive status prediction accuracy. These results highlight the potential of the proposed method to alleviate data scarcity in bioacoustics and support intelligent monitoring in smart beekeeping, with broader applicability to other ecological and agricultural domains.

## 1. Introduction

Bioacoustic signals convey critical information about behavior, health, and interactions of living organisms with the environment [1]. With recent advancements in AI, these signals have become increasingly important in applications such as ecological monitoring, medicine, and agriculture [2]. A key challenge is the scarcity of event-specific sounds, where the acoustic signatures are essential for identifying and distinguishing particular biological events [3]. Bioacoustic signals are comprised of unique characteristics, including temporal, spectral, and structural properties, which help to extract meaningful insights for real-world applications. There are various categories of bioacoustics; however, this study focuses explicitly on Bee Bioacoustics due to its vital significance in sustainable pollination, ecosystem health, and agricultural productivity.

Bee bioacoustics is particularly interesting because it helps to improve sustainable pollination, and that is fundamental for ensuring global food security [2]. Recent studies have shown that bee bioacoustics can support efficient hive and behavioural management by detecting changes in sound patterns associated with stress, swarming, or queenlessness [3]. Distinct acoustic signatures have been observed for key colony events such as queen presence, queen absence, and swarming, each carrying important implications for colony stability and productivity. For example, queen-less hives represent characteristic shifts in acoustic patterns that can be detected well before visual inspection, while swarming events generate specific pre-swarm signals that serve as early indicators for imminent colony reproduction and potential hive division [4]. This represents bee bioacoustics as an active area of research, attracting attention across agriculture, AI, and environmental monitoring domains. This research focuses specifically on bee bioacoustics as a case study, due to its critical ecological and agricultural importance. However, analyzing bee Bioacoustic signals comes with unique technical challenges.

With recent advancements in AI and ML technologies, Bioacoustic signals are now being effectively analyzed and applied across a wide range of domains such as apiculture, wildlife conservation, medical diagnosis, and more [2]. Developing robust machine learning models for bioacoustics analysis requires large volumes of clean, representative data, which is extremely challenging to obtain. Collecting sufficient high-quality bioacoustics signals is difficult due to contamination from environmental noise such as wind or water sounds, the labor-intensive nature of data collection, and the high associated costs [5,6,7]. This scarcity of quality data presents a significant bottleneck for both research and practical deployment of bioacoustics systems. A key challenge is the scarcity of event-specific sounds, where the acoustic signature is essential for identifying and distinguishing particular biological events. These challenges are equally evident in Bee Bioacoustics, where data quality and sufficient data availability are critical factors for advancing research and applications in this field [7].

A practical solution is to generate synthetic bioacoustics datasets [7] that are reliable, balanced, and capture event-specific characteristics. This is especially valuable for rare or hard-to-capture events that are essential for intelligent monitoring systems. While Generative Adversarial Networks (GANs) have shown potential in audio synthesis, they often fail to fully capture the long-term temporal dependencies inherent in bee bioacoustics. This limitation becomes particularly critical when generating event-specific sounds, where acoustic signals carry the essential signatures needed to accurately identify and distinguish events.

### 1.1. Related Work and Comparison with Existing Methods

Synthetic bioacoustic data generation has gained increasing attention due to the critical challenges associated with collecting large, high-quality real-world recordings, particularly for ecologically sensitive species such as honeybees. Traditional data generation techniques such as rule-based [8], random sampling [8], statistical [8] and parametric methods [8] are often use explicit assumption about data distributions to generate synthetic data. They are most suited for simple and structured data with low variability. However, they are insufficient for modeling the complex spectral and temporal patterns inherent in bioacoustic signals.

As a result, machine learning–based generative models have emerged as a promising alternative [3]. Compared to standard or traditional synthetic data generation approaches, ML-based approaches demonstrate great potential in capturing complex patterns and relationships in both structured and unstructured data [8]. While conventional data generation approaches are limited to simple and structured data types, ML-based approaches have an enhanced ability to learn underlying distribution and complex relationships directly from real-world data to model them more accurately [9]. Rather than relying solely on handcrafted rules or predefined statistical models, ML-based generative models can capture intricate interdependencies that standard techniques often overlook [10]. Due to this capability, ML-based synthetic data generation approaches have made remarkable progress across multiple domains such as computer vision, speech generation, natural language processing, healthcare, finance, bioacoustics, and many others, where data diversity is crucial [11]. Among ML models, Large Language Models (LLMs), Variational Autoencoders (VAEs), Autoregressive models, and Diffusion models have been explored for audio and time-series synthesis.

Large Language Models (LLMs) are designed to model sequential token dependencies and semantic representations while learning contextual dependencies of text data [12]. This design allows the model to capture semantic patterns with logical reasoning within a sequence and that makes it more effective for generating coherent and contextually accurate human-like text [13]. LLMs can create artificial but useful data to enhance or replace small real-world datasets [12]. Based on the research, the effectiveness of synthetic data generated by LLMs is negatively impacted by subjectivity, such as emotions and personal perspectives, and biases in training data [14]. Although LLMs are highly effective in text generation, they are less suitable for handling unstructured or complex data such as images, audio, or bioacoustic data.

Variational Autoencoders (VAEs) consist of two feed-forward neural networks named the encoder and the decoder, which work together to learn the underlying distribution of the input data [15]. The probabilistic modeling of latent variables allows the VAE to capture variability and uncertainty in the data, which enables the generation of diverse synthetic samples. VAEs are widely used in synthetic tabular data generation [16] and image synthesis, although their performance is generally less effective [17]. Despite the usages, one of the main drawbacks of VAEs is their high computational cost and longer processing time [18]. VAEs are less effective in modeling complex data distributions and lack the ability to handle high-dimensional datasets such as images and audio effectively [15]. Their usage in the bioacoustics domain is limited, which could be due to their difficulty in preserving fine details of audio data.

Autoregressive Models (ARMs) are made to produce data sequentially by predicting elements based on the previously generated elements through learned conditional dependencies of the input [19]. This iterative process enables capturing sequential patterns in the input sequence effectively. ARMs have been successfully applied to generating sequential data such as time-series data, tabular data, audio sequences, images, and videos [19]. Despite the benefits, ARMs have several limitations, including computational expense for long sequences, decreased output quality due to early prediction errors, and less realistic output compared to GAN-based models [19]. These limitations make ARMs less suitable for high-fidelity audio generation, such as bioacoustics signals.

Diffusion Models (DMs) are based on iterative denoising diffusion processes, where the model gradually transforms structured data into noise and then reconstructs it back to realistic samples by reversing the noise process [19]. This process helps to avoid issues such as mode collapse and generate quality outputs [19]. Diffusion models have achieved remarkable success in generating high-dimensional, structured data, particularly in image synthesis, video generation, tabular data generation, and 3D modeling [19,20,21]. While diffusion models have proven highly successful in visual and structured data domains, their usage for bioacoustic signal generation is impractical [19].

However, these approaches present notable limitations in the bioacoustics domain. Consequently, these models are less practical for realistic bee bioacoustic signal synthesis. Hence we explored Generative Adversarial Networks (GANs) for synthetic data generation.

Generative Adversarial Networks (GANs) are the most powerful and widely used machine learning models for synthetic data generation, and they have become popular for generating both static and unstructured data, such as image data, tabular data, voice data, time-series data, financial-related data, and some bioacoustic signals. There are many improved versions of GANs, such as Conditional GAN (cGAN), Tabular GAN (TGAN), Conditional Tabular GAN (CTGAN) [22], Deep Convolutional GAN (DCGAN), Multivariate Time series GAN (MTS-GAN), and many others that have been developed to enhance their performance for different applications [8,23]. High quality and flexibility are key advantages of GANs. They can produce realistic and reliable data while effectively modeling complex and high-dimensional data distributions such as images and audio [8]. This breakthrough development of GANs has positively impacted various sectors such as entertainment, finance, healthcare, and research [24]. For example, in healthcare, high-quality synthetic images help improve disease diagnosis, and in entertainment, synthetic music and sound generation improve content creation with advanced visual effects. Though there has been wide success in static data, such as image synthesis using GANs, there are only a few applications in audio data generation. In the bioacoustics domain, certain variants of GANs have been used for bee data classification, augmentation, and noise reduction [25]. Their ability to model complex characteristics of bioacoustic signals have made them particularly suitable for data augmentation tasks [25]. GANs can produce high-quality outputs and achieve good performance with high accuracy. However, GANs also present with several limitations, such as, training instability, mode collapse (limited diversity in data), longer training times with increased computational complexity and difficulty capturing temporal dependencies [23].

Despite some limitations, the literature on synthetic data generation shows that GANs are more popular and powerful than other ML-based approaches. Given that this research focuses on bee bioacoustics, further exploration has been conducted on specific GAN variations, such as WaveGAN [26], SpecGAN [27], and StyleGAN [28], which are specialized in audio and bioacoustic signal generation. WaveGAN is an extension of DCGAN designed for unsupervised generation of 1D raw audio waveforms [27]. It is one of the earliest GAN-based approaches for audio synthesis. By directly modeling temporal audio signals, WaveGAN achieves stable training and produces high-quality, realistic audio at high speed, with successful applications in marine bioacoustics, bird sounds, and creative multimedia. However, it struggles to preserve dynamic behavior over time and is limited to short output durations. In contrast, spectrogram-based GAN models (SpecGAN and StyleGAN) represent audio as 2D time–frequency images and they provide more stable training and effective learning of spectral structures, particularly for music, speech, and whale vocalization synthesis [28]. Despite these advantages, such models are computationally expensive, fail to capture fine-grained long-term temporal dependencies, and suffer information loss during spectrogram-to-waveform reconstruction.

To address temporal modeling limitations in standard GANs and their variations, hybrid approaches combining GANs with probabilistic sequential models have been explored. Markov Chain Generative Adversarial Networks (MCGANs) integrate the adversarial learning capability of GANs with the probabilistic transition modeling of Markov Chains. Such models have demonstrated improved stability and temporal consistency in domains including physics, multivariate time-series synthesis, agriculture, and medical diagnostics. The Markov property [29] enables effective modeling of sequential dependencies by conditioning each generated state on the previous one, thereby preserving dynamic behavior that standard GANs often fail to capture.

### 1.2. Problem Formulation and Summary of Contribution

The fundamental problem addressed in this study spans multiple domains–bioacoustics, signal processing, probabilistic modeling, and machine learning. Bee colonies display event-specific acoustic patterns that reflect internal hive states such as the queen bee’s presence or absence. These acoustic patterns are temporally structured and impacted by environmental and behavioural dynamics, hence it is challenging to model them using conventional deterministic approaches.

From a signal processing standpoint, bee bioacoustic signals can be modeled as time-domain signals with event-dependent spectral and temporal features within a limited frequency range. However, there are issues with data scarcity and class imbalance that impede robust statistical learning because real-world recordings are frequently scarce, tainted by noise, and unevenly distributed across events.

From a machine learning perspective, the task can be formulated as learning an underlying probability distribution that governs event-specific bee acoustic signals, such that new samples drawn from this distribution preserve both global spectral properties and local temporal dynamics. Generative Adversarial Networks (GANs), particularly WaveGAN, provide a data-driven mechanism for learning high-dimensional waveform distributions directly from raw audio and synthesize them. However, standard GAN-based approaches do not support to preserve temporal dependency constraints that are critical for biological signal realism.

To address the limitations of existing synthetic data generation methods in bee bioacoustics, this study explores the integration of Markov Chains (MC) as a refinement stage based on Markov Chain theory. The refinement process formulates synthetic signal generation as a stochastic transition process, where each generated signal evolves toward the target real-data distribution through probabilistic acceptance governed by the Metropolis–Hastings algorithm. This MC process helps us to synthesize event-specific Bioacoustic signals generated by a specialized GAN variant, WaveGAN. MC is a well-established mathematical framework widely used in predictive modeling [30]. It has also been successfully integrated with GANs to better handle high-dimensional data and produce more realistic and reliable outputs [29,31]. However, to the best of our knowledge, the integration of MC with the WaveGAN approach remains underexplored in the context of Bee Bioacoustic signal generation. In this work, we aim to bridge this gap by addressing the challenges posed by limited high-quality datasets in real-world bee bioacoustics intelligence applications. Overall, considering the motivation outlined above, this study proposes a novel hybrid framework, termed MCWaveGAN, which extends the conventional WaveGAN architecture by introducing a Markov Chain-based refinement stage implemented via the Metropolis–Hastings algorithm to generate synthetic bee bioacoustic signals. Through this approach, this research focuses on addressing one of the main challenges of Bee Bioacoustics research and applications; the lack of quality and sufficient real-world data by generating synthetic signals. Unlike prior GAN-based audio synthesis approaches, the proposed method explicitly enforces temporal coherence and spectral consistency by probabilistically refining generated signals toward the distribution of real bee bioacoustic features. To the best of our knowledge, this is the first work to apply Markov Chain refinement to WaveGAN for event-specific bee bioacoustic signal generation, enabling significantly improved realism and downstream classification performance.

In this research, we design and develop a robust generative framework for synthetic bee bioacoustic signal generation by integrating WaveGAN with a Markov Chain–based refinement process. The proposed model aims to synthesize realistic, event-specific bee acoustic signals by effectively capturing both spectral characteristics and temporal dynamics inherent in real hive recordings. Using the developed framework, a synthetic bee bioacoustic signal dataset is produced and systematically evaluated to assess its realism and utility. The validation is conducted through quantitative measures, including classification performance using real test data, as well as qualitative analyses. These qualitative evaluations focus on spectral and statistical similarity between the generated synthetic signals and real bee sounds, ensuring that the synthesized data closely reflects the acoustic properties of natural bee bioacoustic events.

## 2. Materials and Methods

In Bee Bioacoustics research, scarcity and noise of real-world datasets are a critical challenge when developing and training robust machine learning models. To overcome it, this study proposes a framework that can generate synthetic event-specific bee bioacoustic signals that are close to real bee signals. Specifically, this framework aims to produce synthetic recordings representing queen-present and queen-absent hive conditions.

**Figure 1:**
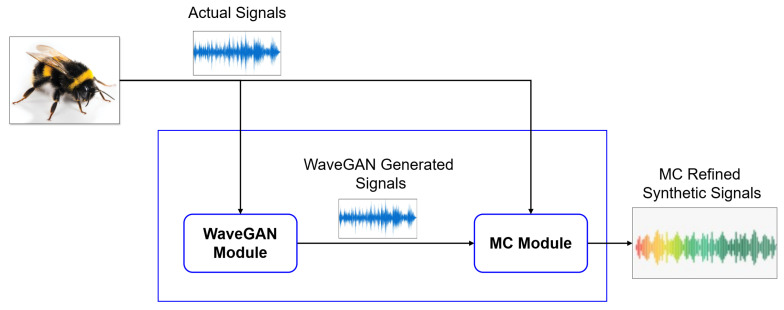
High-level architecture of the proposed MCWaveGAN Framework for synthetic bee bioacoustic signal generation.

The proposed model consists of two main components: a WaveGAN module, which generates initial synthetic signals, and a Markov Chain module, which refines them to preserve temporal dependencies and acoustic fidelity. The proposed hybrid approach first uses the WaveGAN module to generate bee signals, which are then refined by Markov Chain module as depicted in Figure 1. In addition, we include a lightweight classification stage, making the framework a complete pipeline for event-specific bioacoustic analysis.

### 2.1. WaveGAN Module

First proposed by Goodfellow et al. in 2014 [32], GANs are regarded as one of the most promising generative models for producing data. The ability of GANs to learn from real data has made them superior in synthetic data generation compared to traditional and other ML-based approaches. Today, GANs are most popular due to their widespread applicability and exceptional capability to model complex and high-dimensional data [33]. GAN-based models consist of a *generator* (*G*), which produces synthetic samples, and a *discriminator* (*D*), which distinguishes between real and generated data. The adversarial training process between *G* and *D* enables GANs to learn the underlying distribution of complex datasets.

In our proposed framework, the WaveGAN Module employs WaveGAN as the baseline model for generating synthetic bioacoustic signals [27]. It is recognized as the first GAN-based model designed for unsupervised raw audio waveform generation [27]. Its architecture was adopted from DCGAN, originally designed for image synthesis [27]. Technically, WaveGAN adapts the DCGAN architecture by reshaping convolutional layers to directly process audio waveforms [34]. The generator of WaveGAN produces floating-point audio signals, while the discriminator employs 1D convolutions to distinguish between real and synthetic waveforms. Training stability is enhanced through the use of Wasserstein GAN with Gradient Penalty (WGAN-GP) [27]. WaveGAN has demonstrated promising results across various audio synthesis domains. It has been applied to speech generation (e.g., spoken digits) [35] from raw audio waveform and to generate drum beats and instrumental sound effects for the entertainment domain [36]. In the bioacoustics domain, WaveGAN has been used to generate bird calls and dolphin sounds, demonstrating its capability to model natural acoustic patterns [26]. However, the review of the literature indicates that the application or research of WaveGAN to bee bioacoustics has not yet been explored.

Although WaveGAN can capture general acoustic properties, it struggles to preserve essential event-specific sound characteristics, which are critical for identifying acoustic signatures. These characteristics are particularly critical for downstream classification tasks where the goal is to identify event-specific sounds (e.g., distinguishing hive events). To overcome these limitations, we introduce a Markov Chain Module, which refines the WaveGAN outputs to better capture event-related signal characteristics.

### 2.2. Markov Chain Module

A Markov Chain is a mathematical model to represent a randomly changing system and its state transitions according to a set of probabilistic rules where the future states only depend on the current state and not on the sequence of past states (Markov property) [30]. This makes Markov Chains particularly suitable for modeling sequential and time-dependent signals, such as audio. In the context of bee bioacoustic signals, this property can support gradual refinement of synthetic audio features while preserving temporal continuity and avoiding abrupt transitions that may introduce acoustic artifacts. In recent studies, integration of Markov Chain process with GANs has shown great advancements in various domains, particularly in handling sequential and time-dependent data. MCGAN represents a combination of the strengths of the Markov Chain process with the adversarial learning capability of GANs. One of the most prominent applications of MCGAN is to solve Bayesian inverse problems in physics and engineering [29], and it has shown a great performance compared to traditional methods. There, MCGAN generates high-quality outputs from complex, high-dimensional input data with higher accuracy and faster computational efficiency [29]. Incorporation of the MC process has helped to improve convergence and produce stable results.

Since WaveGAN generation does not guarantee to preserve temporal dynamics and other finer details of the original data, the MC module is used to address that limitation in this proposed method. The key idea behind MC is to design a process that moves step by step between samples, where each sample depends on the previous one. This sequential progression allows the target distribution to be accurately represented [37]. Among the available implementations, the Metropolis–Hastings (MH) algorithm [37] is the most widely adopted, and it is integrated into our MC module. The MH algorithm builds a Markov Chain whose stationary distribution matches a target probability distribution defined by a Gaussian prior learned from real bee bioacoustic features. At every step the algorithm proposes a new candidate state and either accepts or rejects it. This acceptance mechanism allows the refinement process to favor transitions that improve alignment with real acoustic characteristics while still permitting stochastic exploration of the feature space. As a result, the MH-based Markov Chain refinement enhances spectral consistency, temporal smoothness, and overall realism of the WaveGAN-generated bee bioacoustic signals.

Once the WaveGAN converges, candidate features v are drawn from the pool of the generated signals, and the MH algorithm decides whether to accept or reject them. During this process, a transition vector $\theta_{\mathrm{prop}}$ is defined as [31]:

(1) $$\theta_{\mathrm{prop}}=\left(1-\beta \right)\left(v-s_{t}\right)+\beta \left(v-r_{t}\right)$$

where,

$\theta_{\mathrm{prop}}$: Proposed transition vector generated by the Markov Chain refinement step.
β: Balance parameter controlling the trade-off between similarity to real signals and smooth transition from the current synthetic signal, where 0≤β≤1.
*v*: Candidate feature vector sampled from the pool of WaveGAN-generated synthetic bee audio signals.
$s_{t}$: Current synthetic bee bioacoustic signal feature vector at iteration *t* of the Markov Chain process.
$r_{t}$: Corresponding real bee bioacoustic signal feature vector at iteration *t* drawn from the real dataset.

The acceptance probability is given by α as defined in the Equation (2) [31]:

(2) $$\alpha =\min\left(1,\frac{p(\theta_{\mathrm{prop}})}{p(\theta_{t})}\right)$$

where,

α: Acceptance probability used in the Metropolis–Hastings algorithm to decide whether the proposed transition is accepted.
$p(\theta_{\mathrm{prop}})$: Probability density of the proposed transition vector under the assumed Gaussian prior distribution.
$p(\theta_{t})$: Probability density of the current state vector under the same Gaussian prior distribution.
$\theta_{t}$: Current state of the Markov Chain at iteration *t*.
min(·): Function that ensures the acceptance probability is bounded within the interval [0,1].

Each state θ corresponds to a feature-level representation of an audio signal segment, encoding essential acoustic characteristics such as amplitude variation, spectral energy distribution, and short-term temporal structure rather than raw waveform samples. The probability density function p(·) represents the Gaussian prior [38] that models the transition probabilities of real audio features. A random number *u* from a uniform distribution is taken to determine the acceptance. If u<α, the candidate is accepted. If u≥α, no action is taken. Accepted samples are further stabilized using Exponential Moving Average (EMA) smoothing [39] to reduce sudden jumps between features. From a signal-processing perspective, this acceptance mechanism biases the refinement process toward feature configurations that preserve the spectral and temporal characteristics of real bee sounds while maintaining stochastic variability. As a result, Equation (2) enforces both temporal smoothness and acoustic fidelity during the Markov Chain refinement of WaveGAN-generated signals.

This proposed **MCWaveGAN** model integrates the strengths of WaveGAN and Markov Chain process to generate realistic synthetic bee bioacoustic signals. It combines WaveGAN’s capability to learn raw audio waveforms with the temporal modeling power of a Markov Chain to produce signals that not only replicate the natural acoustic patterns but also preserve event-specific characteristics and essential temporal features. It is expected that this approach enhances the realism of generated signals to support advanced research and applications in bee bioacoustics and ecological monitoring.

### 2.3. LDA-SVM Evaluation Framework

We consider a simple classification task to evaluate the usefulness of the synthetically generated data. The goal is to classify audio samples into three event categories. (e.g., *QueenPresent*, *QueenAbsent*, and *NoBee*). To achieve this, each audio audio signal is first transformed into Mel-Frequency Cepstral Coefficients (MFCCs) [40], where. MFCC serve as the primary acoustic feature representation for the subsequent LDA-SVM classification. Each 1-second audio segment is represented using 20 MFCC coefficients and that effectively capture the perceptual and spectral characteristics of bee sounds and helped to distinguish between *QueenPresent* and *QueenAbsent* bee hive conditions. This process ensured consistent feature representation across all samples while maintaining robustness to noise and minor variations in signal amplitude.

To reduce dimensionality and highlight the discriminative structure of the data, we apply Linear Discriminant Analysis (LDA). Since the classification task involves three distinct classes (C=3), LDA projects the MFCC features onto a 2-dimensional subspace (i.e., C−1 components). This transformation simplifies the feature space and makes the classification task more tractable, enabling a clear visualization of class separation. For the classifier, we adopt a simple SVM model trained on the LDA-transformed features.

## 3. Experiments and Results

### 3.1. Dataset Description and Pre-Processing

**Figure 2:**
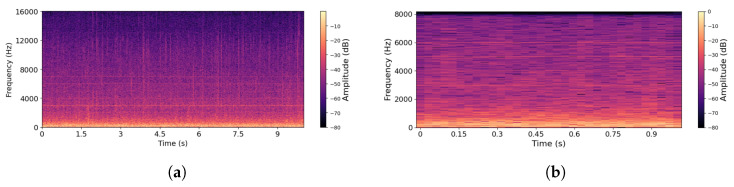
Spectrograms of *QueenPresent* bee signals: (**a**) before processing and (**b**) after processing.

**Figure 3:**
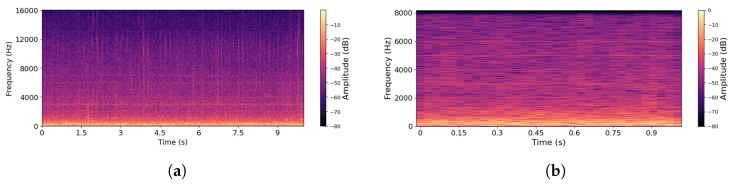
Spectrograms of *QueenAbsent* Bee signals: (**a**) before processing and (**b**) after processing.

In order to demonstrate the validity of the proposed methods, we apply them to a bee bioacoustics dataset [41,42] that includes three distinct events with their acoustic signatures: *QueenPresent*, *QueenAbsent*, and *NoBee*. Each sound recording in the dataset has a duration of 10 s, with most of the acoustic energy concentrated below 2 kHz. In total, the dataset provides 4000 *QueenPresent* and 2000 *QueenAbsent* recordings at an original sampling rate of 32 kHz. In addition to the bee categories, a *NoBee* dataset was included to strengthen validation. Several preprocessing steps were applied to standardize and enhance the dataset. First, the recordings were downsampled to 16,384 Hz and converted to mono. Silence intervals were removed to ensure that each clip starts and ends with an active bee sound. Band-pass filtering (20–2000 Hz) was applied to remove irrelevant frequency components. Finally, the recordings were segmented into 1-second clips, with padding or truncation applied to maintain fixed durations. After processing, the final dataset consisted of 39,838 *QueenPresent*, 19,897 *QueenAbsent*, and 9964 *NoBee* samples. After preprocessing of raw bee audio signals, the spectrograms of *QueenPresent* and *QueenAbsent* samples showed significant improvements in spectral clarity and harmonic structures. Representative spectrograms of both bee-event categories are presented in Figure 2 and Figure 3. Figure 2 illustrates the effect of the preprocessing pipeline on *QueenPresent* bee signals using time–frequency representations. Figure 2a shows the spectrogram of a raw signal before processing, where acoustic energy is spread across a wider frequency range and partially obscured by background noise and low-energy components. In this representation, harmonic structures associated with sustained bee activity are less distinct, and transient noise introduces irregular spectral patterns. In contrast, Figure 2b presents the spectrogram after preprocessing, where the application of band-pass filtering and silence removal results in clearer temporal continuity, reduced noise, and a stronger concentration of energy within the biologically relevant low-frequency bands. The processed signal exhibits more stable harmonic patterns over time, which are characteristic of queen-present hive conditions and are essential for reliable feature extraction and downstream modeling. Similarly, Figure 3a,b show the *QueenAbsent* bee signals before and after preprocessing, respectively, where the same steps reduce background noise and clarify the spectral structure, making the acoustic differences associated with the absence of the queen easier to observe.

Depending on the objective of the experiment, such as pretraining, synthetic sample generation, or classification performance evaluation, appropriate portions of the dataset were utilized. This approach ensured that each experiment setup was optimized for its intended usage while maintaining methodological consistency across the study.

### 3.2. MCWaveGAN Outperforms WaveGAN in Accuracy and Acoustic Realism

The first experimental claim evaluates the effectiveness of the proposed MCWaveGAN model compared to the WaveGAN alone in generating realistic bee audio signals. This analysis focused on both event-specific bee sound categories: *QueenPresent* bee and *QueenAbsent* bee.

#### 3.2.1. WaveGAN Generation

The *QueenPresent* subset of the processed dataset (15,942 samples) was first used to train the WaveGAN model for 120,000 iterations with a batch size of 64, in order to generate synthetic *QueenPresent* samples. The model producesC 1-second audio segments at a sampling rate of 16,384 Hz, generating 64 samples and saving both checkpoints and outputs every 1000 iterations. After training, the generated samples from each checkpoint were evaluated using an LDA-SVM model (trained on real data) to determine the point of convergence. At iteration 48,000, the WaveGAN achieved its best performance, producing the highest proportion of correctly classified *QueenPresent* samples, with 45 out of 64 correctly identified. To further assess temporal dynamics, 20,000 synthetic samples were generated from this converged model and classified using the LDA-SVM classifier. With WaveGAN alone, only 59.8% of the generated samples were correctly classified as *QueenPresent*, while 40.1% were misclassified. The 59.8% accuracy doesn’t necessarily mean the WaveGAN outputs are completely poor, as it could also reflect that the classifier (LDA-SVM trained only on real data) has not generalized well to the distribution of the synthetic data. However, as later demonstrated through LDA projections of the generated signals, these misclassifications are primarily due to poor data generation by WaveGAN.

The same procedure was then applied to the 15,941 *QueenAbsent* samples and 20,000 synthetic *QueenAbsent* samples were generated using the WaveGAN. Classification using the pre-trained LDA-SVM model revealed that out of a total of 20,000 samples, 85.8% were classified as *QueenAbsent*, while the remaining 14.2% were misclassified into the other two categories.

#### 3.2.2. MC-Refined WaveGAN Generation (MCWaveGAN)

In our proposed MCWaveGAN model, the first phase consists of the WaveGAN module to generate synthetic bee signals, and the second phase involves the MC refinement module to address the limitations of the WaveGAN output. The WaveGAN-generated samples were fed into this MC module, together with the corresponding real samples, for refinement using the Metropolis-Hastings algorithm with varying β parameters to balance fidelity and diversity. This process was configured to produce 20,000 audio clips per class (*QueenPresent* and *QueenAbsent*), each 1 s in duration and sampled at 16,384 Hz. For *QueenPresent* type, the optimal performance was achieved with $\beta_{\mathrm{opt}}$= 0.01, with 99.9% of the refined samples correctly classified as *QueenPresent*, as shown in Table 1. These results confirm that the MC refinement process substantially enhances the realism and class fidelity of the generated *QueenPresent* bee signals.

*QueenAbsent* experiment runs the same MC refinement process for the WaveGAN-generated *QueenAbsent* samples with multiple β values and $\beta_{\mathrm{opt}}$ = 0.9 achieved the most stable convergence and discriminative results. When validating MC refined samples with the pre-trained LDA-SVM model, it classified 19,911 out of 20,000 samples as *QueenAbsent* while 89 as *NoBee*, and none as *QueenPresent* as shown in the Table 2. This represents a remarkable refinement of the class boundaries compared to the WaveGAN output, with 99.6% samples being classified as *QueenAbsent*. Hence, it suggests that the Markov Chain refinement effectively captures and preserves realistic features of real bee data.

The MH algorithm employed with different β values controls the trade-off between retaining the characteristics of the real samples and maintaining smooth transitions in the synthetic data. The refined samples were subsequently validated using the LDA-SVM model (trained on real data) for classification accuracy. After refinement with the MC module, performance improved significantly.

**Figure 4:**
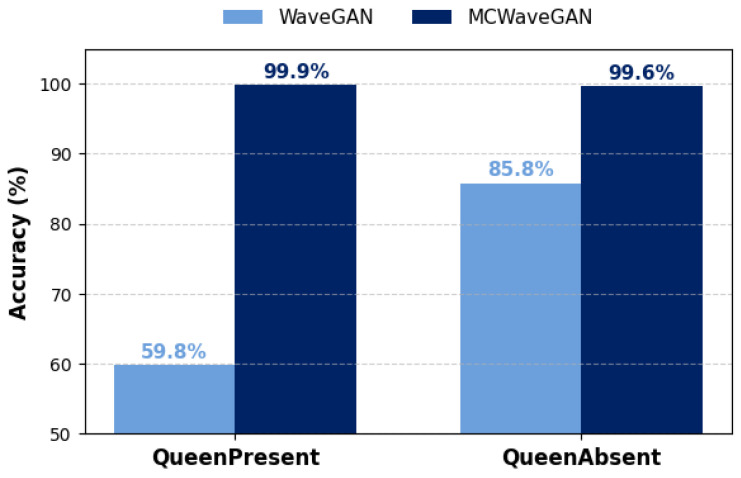
Comparison of classification accuracy for synthetic bee signals generated by proposed MCWaveGAN and WaveGAN alone for QueenPresent and QueenAbsent signals.

Table 3 and Figure 4 illustrate the comparison of classification accuracy between synthetic bee signals generated after MC refinement and those produced by the WaveGAN model. Table 3 summarizes the LDA-SVM classification results for synthetic bee bioacoustic signals generated using WaveGAN alone and after refinement with the proposed Markov Chain process (MCWaveGAN). For the *QueenPresent* class, WaveGAN-generated samples exhibit substantial misclassifications, with only 59.8% of the samples correctly identified, indicating that WaveGAN struggles to preserve event-specific acoustic characteristics. After MC refinement with an optimal value of β=0.01, the classification accuracy improves significantly to 99.9%, with almost all samples correctly classified as *QueenPresent*. A similar trend is observed for the *QueenAbsent* class. While WaveGAN-generated *QueenAbsent* samples achieve an accuracy of 85.8%, a notable proportion of samples are misclassified into other categories. After MC refinement using β=0.9, the correct classification rate increases to 99.6%, demonstrating that the MC refinement stage effectively enhances the temporal coherence and spectral consistency of the generated signals. Overall, the results in Table 3 confirm that the proposed MCWaveGAN model substantially outperforms WaveGAN alone in generating acoustically realistic and event-discriminative bee bioacoustic signals. In both cases, only less than 1% were incorrectly classified. The results clearly demonstrate that the combined MCWaveGAN model substantially outperforms WaveGAN alone in generating realistic bee sound signals. Further, this indicates that the MC refinement achieves a strong balance between maintaining the acoustic fidelity of real bee signals and ensuring smooth transitions in the synthetic data.

#### 3.2.3. Statistical Analysis

Statistical analysis compare frequency distribution, amplitude distribution, and spectral centroid for three randomly selected samples of the actual and generated data (for the *QueenPresent* and *QueenAbsent* classes). These characteristics are not features used in the AI models, but are included here solely for visualization and comparison. As shown, the MC-refined samples (green) align more closely with the distribution of real signals (blue) than those generated by WaveGAN alone (orange). This indicates that the MC refinement step reduces the divergence observed in WaveGAN outputs, resulting in synthetic signals whose observable acoustic behavior is more consistent with real bee sounds.

**Figure 5:**
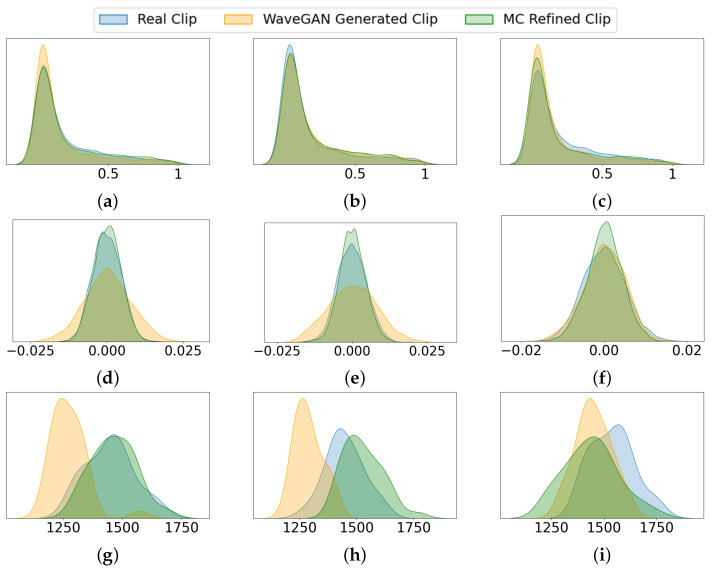
Comparative analysis of acoustic characteristics for actual, WaveGAN-generated, and MC-refined *QueenPresent* samples. Panels (**a**–**c**) show normalized frequency probability distributions, (**d**–**f**) amplitude probability distributions, and (**g**–**i**) spectral centroid probability distributions for three representative sample pairs.

**Figure 6:**
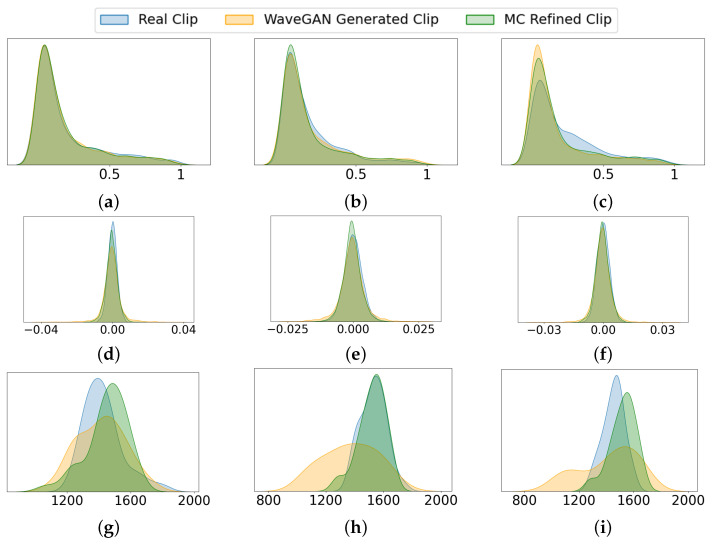
Comparative analysis of acoustic charactristics for Real, WaveGAN-generated, and MC-refined *QueenAbsent* samples. Panels (**a**–**c**) show normalized frequency probability distributions, (**d**–**f**) amplitude probability distributions, and (**g**–**i)** spectral centroid probability distributions for three representative sample pairs.

Figure 5 presents a comparative analysis of acoustic characteristics for real, WaveGAN-generated, and MC-refined *QueenPresent* bee signals using three representative sample pairs. Panels (a–c) illustrate the normalized frequency probability distributions, where the MC-refined samples (green) closely follow the distribution of real signals (blue), indicating improved preservation of dominant frequency components associated with queen-present hive activity. In contrast, WaveGAN-generated samples (orange) exhibit broader and less consistent frequency distributions, reflecting spectral deviations from real bee sounds. Panels (d–f) depict the amplitude probability distributions. The MC-refined signals demonstrate amplitude distributions that more closely match those of real signals, suggesting improved modeling of signal energy dynamics and reduced artificial fluctuations. By comparison, WaveGAN-generated samples display higher variance and less stable amplitude behavior. Panels (g–i) show the spectral centroid distributions, where MC refinement significantly reduces the divergence observed in WaveGAN outputs, resulting in centroid distributions that better reflect the temporal evolution and spectral balance of real *QueenPresent* signals. Overall, Figure 5 demonstrates that the MC refinement stage effectively enhances the statistical and spectral realism of the synthetically generated bee bioacoustic signals. Figure 6 presents the same comparative analysis for the *QueenAbsent* class which reflects that the MC-refined signals more closely match the frequency, amplitude, and spectral centroid distributions of real bee sounds than WaveGAN-generated samples.

#### 3.2.4. LDA Analysis

**Figure 7:**
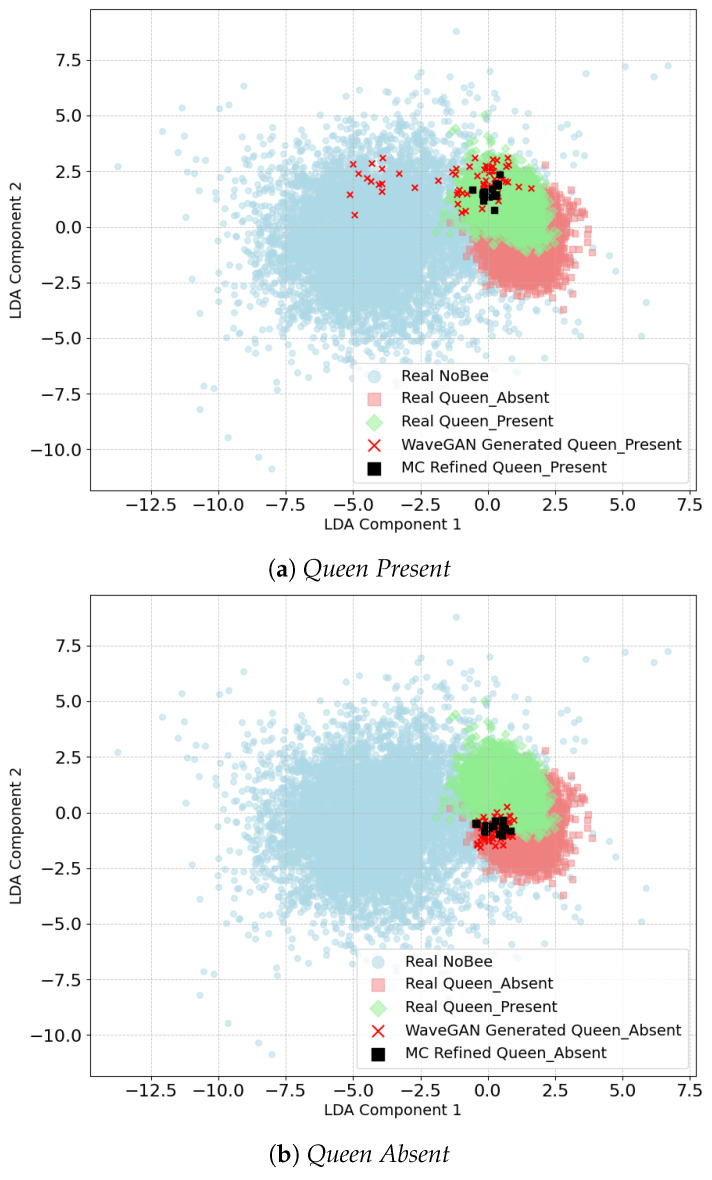
LDA projections of actual bee bioacoustic signals compared with WaveGAN-generated and MC-refined synthetic samples. The plots illustrate how MC refinement shifts synthetic data closer to the real class distribution for both (**a**) *QueenPresent* and (**b**) *QueenAbsent* categories.

LDA projections were used to visually examine the alignment of the synthetic data distributions with the real data. The plot depicted in Figure 7 clearly shows that WaveGAN-generated samples are dispersed while MC-refined samples are tightly clustered around the real *QueenPresent* and *QueenAbsent* regions, respectively. This shift in data distribution illustrates that the MC refinement effectively reduces class overlap and pushes synthetic data close to real data. Hence, it improves realism and enforces structural similarity within the feature space.

#### 3.2.5. Real-Data Evaluation with Augmented Training

We evaluated the impact of synthetic bee signal augmentation by comparing classification performance for both *QueenPresent* and *QueenAbsent* samples generated by WaveGAN alone vs. those refined through the MC process (MCWaveGAN). We define two models using previously generated bee audio samples: Model 1 is trained on real data plus WaveGAN-generated bee signals, and Model 2 is trained on real data plus MCWaveGAN-generated bee signals (i.e., WaveGAN-generated, and then refined by the MC process). Classification performance was evaluated using an LDA–SVM classifier with MFCC-based features and quantified using standard metrics including accuracy, precision, recall, and F1-score, computed on real test data. This experiment was done for both *QueenPresent* and *QueenAbsent* datasets separately.

For *QueenPresent* samples, the classification results shown in Table 4 indicate that MCWaveGAN augmentation outperforms WaveGAN-generated augmentation. While recall remained stable at 98% for both models, the precision increased from 89% to 95%. That indicates a significant reduction in false positives. The F1-score also improved from 93% to 96%, and that reflects the realism improvement of the synthetic *QueenPresent* audio. Furthermore, the overall accuracy increased from 94% in Model 1 to 96% in Model 2, demonstrating the effectiveness of the MC refinement stage in improving classification performance.

A similar experiment was conducted for *QueenAbsent* using WaveGAN and MCWaveGAN generated samples. There, Model 1 was trained on real data combined with WaveGAN-generated *QueenAbsent* samples, while Model 2 was trained on real data augmented with MCWaveGAN-generated *QueenAbsent* samples. The Classification results comparison shown in Table 4 demonstrates that the Model 2 achieves consistent improvement for the *QueenAbsent* as well. There, Model 1 achieved a precision of 94%, recall of 96%, and F1-score of 96%, with overall accuracy of 96%. In contrast, Model 2 improved precision to 97%, accuracy to 97%, F1-score to 97%, while maintaining recall at 96%. These results indicate that the MCWaveGAN-generated synthetic bee bioacoustic signals enhance the classifier’s ability to more accurately distinguish *QueenPresent* and *QueenAbsent* acoustic patterns with higher accuracy.

The comparative evaluation between baseline WaveGAN (Model 1) and the proposed MCWaveGAN (Model 2) clearly demonstrates that integrating the Markov Chain refinement significantly enhances synthetic bee sound generation and classification performance. Both *QueenPresent* and *QueenAbsent* categories in Model 2 outperformed Model 1 across all evaluated metrics. These consistent performance gains highlight the advantage of incorporating temporal dependencies through Markov Chain transitions. The enhanced temporal coherence and smoother spectral continuity produced by MCWaveGAN significantly contributed to generating more realistic bee bioacoustic signals.

### 3.3. MCWaveGAN-Augmented Training Improves Classification Performance

We investigated the effectiveness of synthetic bee bioacoustic signals generated using the proposed MCWaveGAN model in enhancing the learning capability of machine learning models trained on limited and imbalanced real-world data. The occurrence of bee sounds in natural bee-hive environments varies based on the presence or absence of the queen, and that creates naturally unbalanced acoustic conditions. Many recent studies have highlighted the potential of acoustic monitoring for assessing the colony state, particularly for detecting queen presence/absence and swarming preparation, both of which are critically important for colony health and productivity [4,43].

To replicate natural bee hive conditions, an unbalanced dataset was intentionally constructed for both event types. The experiment evaluated whether the proposed MCWaveGAN generated synthetic bee bioacoustic signals of these two event types could augment the real training dataset to improve classification performance. The goal of the experiment was to enhance the model’s sensitivity to rare bee events while maintaining robust performance for automated hive monitoring applications. It is expected to use the proposed MCWaveGAN model to generate the bee bioacoustic signals and overcome the data scarcity challenge for rare hive events while improving the reliability of acoustic classification models. It supports beekeepers in the timely detection of colony conditions. *QueenPresent* related experiment started with only 20% of *QueenPresent* samples and gradually increased its proportion with MCWaveGAN generated samples until matched with *QueenAbsent* samples, eventually the model balance is reached. Conversely, the second experiment began with 20% *QueenAbsent* samples and progressively increased its proportion with synthetically generated samples from MCWaveGAN to equal the *QueenPresent* samples. That helped us to identify the model’s adaptability to changing class distributions and how synthetic bee bioacoustic signals impact the model performance.

#### 3.3.1. QueenPresent Augmentation

For this experiment, we define Model 1 as the baseline model, and it is trained using only real bee audio data, where *QueenPresent* is the minority class. Classification was performed using an LDA–SVM classifier with MFCC-based features, and performance was evaluated on real test data using standard metrics including accuracy, precision, recall, and F1-score. When the Model 1 was trained and classified with the LDA-SVM model, it achieved an overall accuracy of 94.22% along with a precision of 85%, a recall of 89%, and an F1-score of 87% as shown in Table 5. Synthetically generated *QueenPresent* samples from MCWaveGAN (WaveGAN-generated and MC refined) were used to augment Model 1’s training set to evaluate its impact on performance with real test data. The classifier’s performance showed gradual improvement as the dataset approached balance through Model 2 to 4 as depicted in Table 5. As shown in Table 5, the classification scores reflect the improvement in accuracy, precision, recall, and F1-score as the training dataset becomes more balanced. The augmented models demonstrate enhanced stability and improved recognition of the minority class *QueenPresent*. And that reflects that MCWaveGAN-generated samples were both realistic and beneficial for model learning.

#### 3.3.2. QueenAbsent Augmentation

To evaluate the scarcity of *QueenAbsent* data in natural hive environments, the dataset was prepared to reflect the real-world class imbalance. The dataset was divided into *QueenPresent*, *QueenAbsent*, and *NoBee* categories with a 40:20:40 ratio, respectively, in the training set. In this setup, we define the baseline classifier model (Model 1) trained using the real bee data only while keeping the *QueenAbsent* as the minority class. The same LDA–SVM classifier with MFCC-based features and the same evaluation metrics (accuracy, precision, recall, and F1-score) were used for the *QueenAbsent* augmentation experiments, with performance assessed on real test data. The baseline model achieved an overall accuracy of 94.82% with class-specific precision, recall, and F1-score as detailed in the Table 6.

Synthetically generated *QueenAbsent* samples from the MCWaveGAN model were then gradually added to the training set of the Model 1 to evaluate their effect on classification performance. Table 6 presents the classification results. The findings show that progressively augmenting the training set with MCWaveGAN-generated *QueenAbsent* samples led to improved classifier performance in accuracy, precision, recall, and F1-score. These results indicate that the synthetic samples were both realistic and beneficial for enhancing model learning.

**Figure 8:**
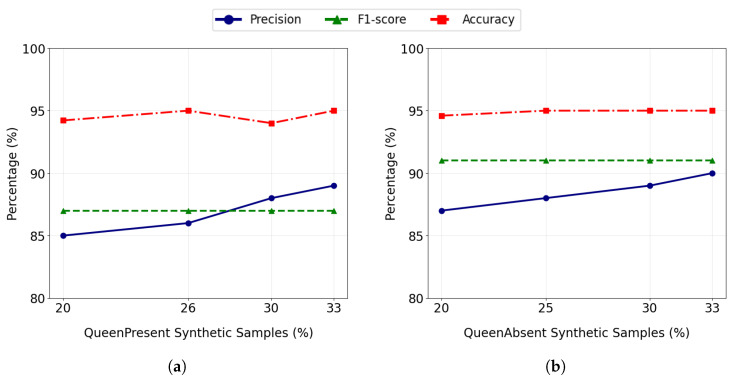
Impact of increasing synthetic samples generated by MCWaveGAN on classifier performance metrics (Precision, F1-score, Accuracy) for (**a**) *QueenPresent* and (**b**) *QueenAbsent* classes, evaluated on real test data.

Figure 8a illustrates the effect of progressively increasing the proportion of MCWaveGAN-generated *QueenPresent* samples in the training set on classifier performance evaluated using real test data. As the number of synthetic *QueenPresent* samples increases, both precision and accuracy exhibit a consistent upward trend with stable F1 score, indicating a reduction in false-positive predictions and improved recognition of the minority class. The overall classification accuracy also improves and stabilizes as the dataset approaches a balanced class distribution, demonstrating that the synthetic signals effectively enhance model generalization. These results confirm that MCWaveGAN-generated samples preserve discriminative acoustic characteristics and contribute positively to classifier learning under data imbalance conditions. Figure 8b shows a similar trend for the *QueenAbsent* class, where increasing the proportion of MCWaveGAN-generated samples in the training set leads to improvements in precision, F1-score, and overall classification accuracy, indicating enhanced robustness and generalization of the classifier under class imbalance.

Overall, the results of this experiment demonstrated that augmenting a real bee audio dataset with synthetic bee signals generated by the proposed MCWaveGAN model can significantly improve machine learning model performance, specifically under conditions of natural data imbalance. It showed noticeable improvements in both overall accuracy and class-level performance. It demonstrated that the synthetic data generated by the MCWaveGAN model can effectively enhance model training when real-world data are limited. This finding provides substantial importance for beekeeping, ecological monitoring, and bee-hive management systems. In real-world conditions, collecting *QueenAbsent* recording is both rare and logistically challenging. Because the occurrence of the queenless state is unpredictable. The ability to synthesize acoustically realistic *QueenAbsent* signals helps researchers to train more robust classification models without requiring extensive real-data collection.

## 4. Discussion

In summary, the results of the study demonstrated that the proposed MCWaveGAN model consistently outperforms the standard WaveGAN in generating realistic bee bioacoustic signals. Statistical analysis illustrated that MC-refined signals are more closely replicate the frequency, amplitude, and spectral centroid distributions of real bee signals compared to WaveGAN outputs. LDA projections further demonstrated that MCWaveGAN-generated samples are tightly clustered around real data, indicating more structural similarity, whereas WaveGAN-generated data are more dispersed. Additionally, real-data performance evaluation with augmented training confirmed that MCWaveGAN augmentation improves classification metrics for *QueenPresent* and *QueenAbsent* synthetic samples with higher precision, recall, F1-score, and overall accuracy than WaveGAN. Overall, these results confirm that the MCWaveGAN model is more effective than the standard GAN (WaveGAN) for realistic synthetic bee signal generation.

Further, augmenting the real bee audio dataset with synthetic bee signals generated by the proposed MCWaveGAN model significantly improves the machine learning model performance, specifically under conditions of natural data imbalance. It showed noticeable improvements in both overall accuracy and class-level performance. It demonstrated that the synthetic data generated by the MCWaveGAN model can effectively enhance model training when real-world data are limited. This finding provides substantial importance for beekeeping, ecological monitoring, and bee-hive management systems. In real-world conditions, collecting *QueenAbsent* recording is both rare and logistically challenging. Because the occurrence of the queenless state is unpredictable. The ability to synthesize acoustically realistic *QueenAbsent* signals helps researchers to train more robust classification models without requiring extensive real-data collection. For real-world hive monitoring applications, improved classification accuracy means that early detection of queenless or hive stress can be achieved with great reliability. This has direct implications for colony health management as it helps beekeepers to take preventive measures before colony collapse occurs. Further, generating balanced and realistic bee bioacoustic signals through the MCWaveGAN model reduces the dependency on manual data collection. This study did not include real-world validation within beehive environments, but the findings suggest that synthetic bee bioacoustic signals generated using the proposed MCWaveGAN model hold strong potential for validation by future researchers. Subsequent research studies could leverage this model to generate synthetic bee bioacoustic signals for practical validations in real beehives. Such that it will give a beneficial contribution to sustainable bee-hive monitoring and preserving bee populations, which is an essential aspect of global agricultural productivity and biodiversity.

In real beehive deployments, it can be challenging to validate the synthetically generated bee bioacoustic signals due to several practical reasons. Acoustics recording may be contaminated by environmental noise like wind, rain and nearby human or machinery activities which can alter the spectral and temporal patterns of these recordings. At the same time, differences in hive structure, seasonal colony behavior, and population dynamics can make signals less consistent and affect classification reliability. These highlight the requirement for noise-robust feature extraction and adaptive modeling when moving from controlled experiments to real bee hive deployments and monitoring.

### Limitations

While the proposed MCWaveGAN framework demonstrates strong performance in generating acoustically realistic and event-discriminative bee bioacoustic signals, there are several limitations.

The datasets utilized in this research experiments is collected using offline, pre-recorded bee bioacoustic datasets under controlled conditions. Although preprocessing steps were applied to reduce environmental noise, real-world hive recordings are subject to additional variability caused by weather conditions, hive structure, sensor placement, and external acoustic interference. As a result, the generalizability of the proposed approach to real-time, in-hive deployment scenarios has not been fully validated.

The proposed framework currently focuses on two primary hive events, QueenPresent and QueenAbsent. Although these events are highly relevant for intelligent hive monitoring, other important colony behaviors, such as swarming, stress responses, or environmental disturbances, were not considered. Extending the model to additional bee events may require further tuning and validation.

In addition, the selection of the Markov Chain balance parameter β was empirically determined for each event class. While optimal values were identified in this study, class-specific parameter tuning may reduce scalability when extending the approach to new datasets or bioacoustic domains. Automated or adaptive parameter selection strategies could improve robustness.

Another limitation of the current study is the absence of behavioral validation at the colony level. Considering the time and scope, this research focuses on passive acoustic analysis only. Future field evaluations should consider the potential effects of continuous acoustic monitoring or experimental playback of synthesized signals on bee behavior, stress responses, and communication dynamics within the hive. Understanding these biological interactions is essential to ensure that synthetic signal-based monitoring remains non-invasive and does not disrupt natural colony functioning.

These limitations suggest several directions for future work, including real-time deployment studies, waveform-level refinement strategies, broader event coverage, and adaptive parameter optimization.

## 5. Conclusions

We have explored and presented a novel approach to generate synthetic bee bioacoustic signals. The proposed model is a hybrid model that combines a WaveGAN and a Markov Chain process. The primary aim of this study was to address the scarcity of high-quality and balanced bee bioacoustic datasets that are required for the AI-driven bee hive monitoring applications and sustainable beekeeping activities. Nowadays, collecting real-world bee bioacoustic data is difficult and expensive. Even when such data is obtained, it is often contaminated by noise from surrounding environmental sounds. This creates a critical challenge in developing robust machine learning models. The integration of the probabilistic modeling capabilities of Markov chains with the generative power of GANs has helped us to refine GAN-generated data to produce synthetic bee bioacoustic signals that closely resemble the original bee signals. Hence, the proposed MCWaveGAN model improved the realism, temporal coherence, and event-specific characteristics of the synthetically generated data. This study explored several research questions related to the generation of synthetic bee bioacoustic data, as outlined in Section 1. First, we explored how limited and noisy bee bioacoustic data can be effectively augmented using generative models, Secondly, we explored how a Markov Chain process can enhance the quality of GAN-generated bee audio signals. The optimal parameters and configurations required to generate the most realistic bee bioacoustic signals are also one of the questions outlined. Lastly, we explored the following question: how realistic are the MCWaveGAN-generated bee signals compared to real bee signals when evaluated quantitatively and qualitatively?

This research systematically addressed the challenge of generating realistic synthetic bee bioacoustic signals by first conducting a comprehensive review of existing synthetic data generation techniques, identifying key limitations in their abilities to preserve the temporal and event-specific characteristics essential for bee communication. This study introduced a novel hybrid architecture, the Markov Chain Wave Generative Adversarial Network (MCWaveGAN), which integrates a WaveGAN-based generative module with a Markov Chain process implemented through the Metropolis–Hastings algorithm to enhance the acoustic realism of generated signals. Experimental evaluations demonstrated that MCWaveGAN consistently outperformed the baseline WaveGAN in both qualitative and quantitative aspects, producing synthetic signals that more closely matched real bee acoustics in terms of frequency, amplitude, and spectral centroid distributions. LDA projections further confirmed that MCWaveGAN-generated samples aligned more closely with real data, indicating stronger structural similarity. Moreover, augmenting real training datasets with MCWaveGAN-generated samples improved classification metrics—including precision, recall, F1-score, and overall accuracy—across both *QueenPresent* and *QueenAbsent* categories, effectively addressing data imbalance caused by rare hive conditions. These findings validate the MCWaveGAN model’s capability to generate high-fidelity synthetic bee bioacoustic data that can serve as a reliable substitute for real recordings. Overall, this study contributes a novel and practical approach to computational bioacoustics, demonstrating how integrating probabilistic temporal modeling with adversarial learning can enhance data realism while supporting sustainable, AI-driven beekeeping and hive monitoring applications by reducing the need for extensive real-world data collection.

While this study and its results have made a successful contribution to generating synthetic bee bioacoustic signals, there are several future directions open for future explorations to advance further and extend this work. One such direction is validating the synthetic bee signal in real beehive environments through controlled field testing. Such evaluations should consider varying environmental noise conditions, sensor placement constraints, and colony-level behavioral responses to assess deployment feasibility, robustness, and long-term practicality of synthetic bee bioacoustic signals in operational hive monitoring systems. This research did not include the testing of synthetically generated *QueenPresent* or *QueenAbsent* sounds in a real beehive to explore their practical usage, as it was beyond the scope of this study. For instance, when a queen leaves a hive, beekeepers often face the risk of colony collapse. In such cases, playing synthetic *QueenPresent* sounds inside the hive could help maintain colony stability until a new queen is introduced and protect the hive. Similarly, synthetic *QueenAbsent* sounds can be used with experimental studies to observe how bees respond when the queen is absent. Such an approach will help the researchers to better understand beehive dynamics and communication patterns that are important for efficient beekeeping.

Generating sound signals of other bioacoustics domains is also a potential future direction. Our study focused on bee bioacoustics, as it is one of the most important domains due to its relevance to apiculture and environmental sustainability; however, the same generative approach can be extended to other bioacoustics domains. Generating realistic synthetic bioacoustic signals for species such as bats, birds, and marine animals would be highly valuable for advancing ecological research and conservation efforts.

## Figures and Tables

**Table 1:** Class distribution of 20,000 *QueenPresent* audio samples generated by WaveGAN and refined using the MC module with different β values, as classified by the LDA–SVM model trained on real bee signals.

	QueenPresent	QueenAbsent	NoBee
WaveGAN Generated	11,963	3	8034
**MC Refined (β=0.01)**	**19,999**	0	1
MC Refined (β=0.05)	19,590	0	410
MC Refined (β=0.1)	3984	0	16,016

**Table 2:** Class distribution of 20,000 *QueenAbsent* audio samples generated by WaveGAN and refined using the MC module with different β values, as classified by the LDA–SVM model trained on real bee signals.

	QueenPresent	QueenAbsent	NoBee
WaveGAN Generated	313	17,159	2528
MC Refined (β=0.01)	155	17,571	2274
MC Refined (β=0.05)	301	17,475	2224
MC Refined (β=0.1)	255	17,887	1858
MC Refined (β=0.5)	180	17,952	1868
**MC Refined (β=0.9)**	0	**19,911**	89

**Table 3:** LDA-SVM classification of WaveGAN-generated and MC-refined bee signals for optimal β values: β=0.01 for *QueenPresent* and β=0.9 for *QueenAbsent*.

	QueenPresent	QueenAbsent	NoBee
WaveGAN-generated *QueenPresent*	11,963 (59.8%)	3	8034
**MC Refined** *QueenPresent* (β=0.01)	**19,999 (99.9%)**	**0**	**1**
WaveGAN-generated *QueenAbsent*	313	17,159 (85.8%)	2528
**MC Refined** *QueenAbsent* (β=0.9)	**0**	**19,911 (99.6%)**	**89**

**Table 4:** Comparison of classification performance for *QueenPresent* and *QueenAbsent* datasets across Model 1 and Model 2.

Category	Metric	Model 1	Model 2
QueenPresent	Precision	89%	95%
	Recall	98%	98%
	F1-score	93%	96%
	Accuracy	94%	96%
QueenAbsent	Precision	94%	97%
	Recall	96%	96%
	F1-score	96%	97%
	Accuracy	96%	97%

**Table 5:** Classification performance on real test data for models trained with varying levels of synthetic *QueenPresent* samples generated by the MCWaveGAN model.

Model	Queen Present	Queen Absent	NoBee	Precision	Recall	F1-Score	Accuracy
Model 1	4K [20%]	8K [40%]	8K [40%]	85%	89%	87%	94.22%
Model 2	5.3K [26%]	8K [40%]	8K [40%]	86%	88%	87%	95%
Model 3	6.8K [30%]	8K [36%]	8K [34%]	88%	85%	87%	94%
Model 4	8K [33.3%]	8K [33.3%]	8K [33.3%]	89%	84%	87%	95%

**Table 6:** Classification performance on real test data for models trained with varying levels of synthetic *QueenAbsent* samples generated by the MCWaveGAN model.

Model	Queen Present	Queen Absent	NoBee	Precision	Recall	F1-Score	Accuracy
Model 1	8K [40%]	4K [20%]	8K [40%]	87%	95%	91%	94.6%
Model 2	8K [37.5%]	5.3K [25%]	8K [37.5%]	88%	93%	91%	95.0%
Model 3	8K [35%]	6.8K [30%]	8K [35%]	89%	92%	91%	95.0%
Model 4	8K [33.3%]	8K [33.3%]	8K [33.3%]	90%	91%	91%	95.0%

## Data Availability

The source code and generated data samples used in this paper are publicly available at: https://github.com/KumuduS/Bioacoustic_MCWaveGAN (accessed on 1 November 2025).

## Abbreviations

The following abbreviations are used in this manuscript:

AI	Artificial Intelligence
ML	Machine Learning
GAN	Generative Adversarial Network
MC	Markov Chain
MCGAN	Markov Chain Generative Adversarial Network
MCWaveGAN	Markov Chain Wave Generative Adversarial Network
SVM	Support Vector Machine
WGAN-GP	Wasserstein Generative Adversarial Network with Gradient Penalty
MH	Metropolis–Hastings
MFCCs	Mel-Frequency Cepstral Coefficients
LDA	Linear Discriminant Analysis
FIR	Finite Impulse Response
